# Different risk factor patterns for adult asthma, rhinitis and eczema: results from West Sweden Asthma Study

**DOI:** 10.1186/s13601-016-0112-0

**Published:** 2016-08-04

**Authors:** Erik P. Rönmark, Linda Ekerljung, Roxana Mincheva, Sigrid Sjölander, Stig Hagstad, Göran Wennergren, Eva Rönmark, Jan Lötvall, Bo Lundbäck

**Affiliations:** 1 Department of Internal Medicine, Krefting Research Centre, Institute of Medicine, Sahlgrenska Academy, University of Gothenburg, Box 424, 405 30 Gothenburg, Sweden; 2 ThermoFisher Scientific, Uppsala, Sweden; 3 Department of Paediatrics, Sahlgrenska Academy, University of Gothenburg, Gothenburg, Sweden; 4 Environmental and Occupational Medicine, The OLIN Unit, Department of Public Health and Clinical Medicine, University of Umeå, Umeå, Sweden

**Keywords:** Asthma, Eczema, Rhinitis, Epidemiology, Prevalence, Population study, Risk factors

## Abstract

**Background:**

Atopic diseases including asthma, rhinitis and eczema have increased in the second half of the past century. This has been well studied among children and adolescents but with the exception of asthma to a much lesser extent in adults. The adult risk factor pattern of atopic diseases, in particular of eczema, and their relation to allergic sensitization are yet to be fully elucidated. Studies among adults that have compared the risk factor pattern for these conditions in the same material are very few. The objective of this study was to compare the risk factor patterns for asthma, rhinitis and eczema in a randomly selected adult population.

**Methods:**

A questionnaire survey on atopic diseases was dispatched by mail to 30,000 randomly selected individuals in West Sweden aged 16–75 years and 62 % participated. A subgroup of 2000 individuals was selected for clinical examinations including blood sampling for specific serum Immunoglobulin E to common airborne allergens and 1172 attended.

**Results:**

The prevalence of current asthma was 11.8 %, current rhinitis 42.8 %, current eczema 13.5 and 2.3 % had all three conditions while 13.9 % had at least two conditions. No mutual risk factor was identified for all three conditions. Allergic sensitization was a strong risk factor for current asthma (OR 4.1 CI 2.7–6.3) and current rhinitis (OR 5.1 CI 3.8–6.9) but not so for current eczema. Obesity was a risk factor for current asthma and current rhinitis, while farm childhood decreased the risk for current asthma and current rhinitis. Occupational exposure to gas dust or fumes and female sex was associated with an increased risk of current asthma and current eczema.

**Conclusions:**

There are different risk factor patterns for asthma, rhinitis and eczema in adults but some risk factors are overlapping between some of the conditions. The effect of mutable risk factors should be assessed further in longitudinal studies.

**Electronic supplementary material:**

The online version of this article (doi:10.1186/s13601-016-0112-0) contains supplementary material, which is available to authorized users.

## Background

The atopic march constitutes the sequential development of atopic diseases [1, 2]. Most often, it starts in early childhood with eczema and progresses with asthma and rhinitis [3–6]. While the exact cause of this temporal pattern remains largely unknown, in recent years the role of the epithelium has gained attention in the pathogenesis of atopic diseases [7, 8]. Filaggrin is an epithelial protein in the epidermis that is responsible for the hydration of stratum corneum [9]. Studies have shown that a mutation in the filaggrin gene predisposes subjects to the development of eczema [10] and mutations in the filaggrin gene are common among subjects with eczema [11]. This impaired skin barrier may lead to allergic sensitization and subsequent development of other atopic diseases such as asthma and rhinitis [12].

Other identified risk factors for atopic diseases in children include exposure to cigarette smoke [13] and family history of atopy [14]. However, the atopic march is mainly related to the incidence of atopic diseases and there is considerate remission of eczema with increasing age [5]. Nevertheless, studies have shown eczema to be common not only among children but also among adults [15–17].

In general, allergic sensitization is associated with asthma, rhinitis and eczema, while the associations with lifestyle and environmental factors show diverging results. Common risk factors for asthma, eczema and rhinitis are infrequently studied simultaneously in adult populations. The aim of this study was to assess individual and common risk factors for asthma, eczema and rhinitis in a randomly selected adult population sample.

## Methods

### Study area and population

**Fig. 1:**
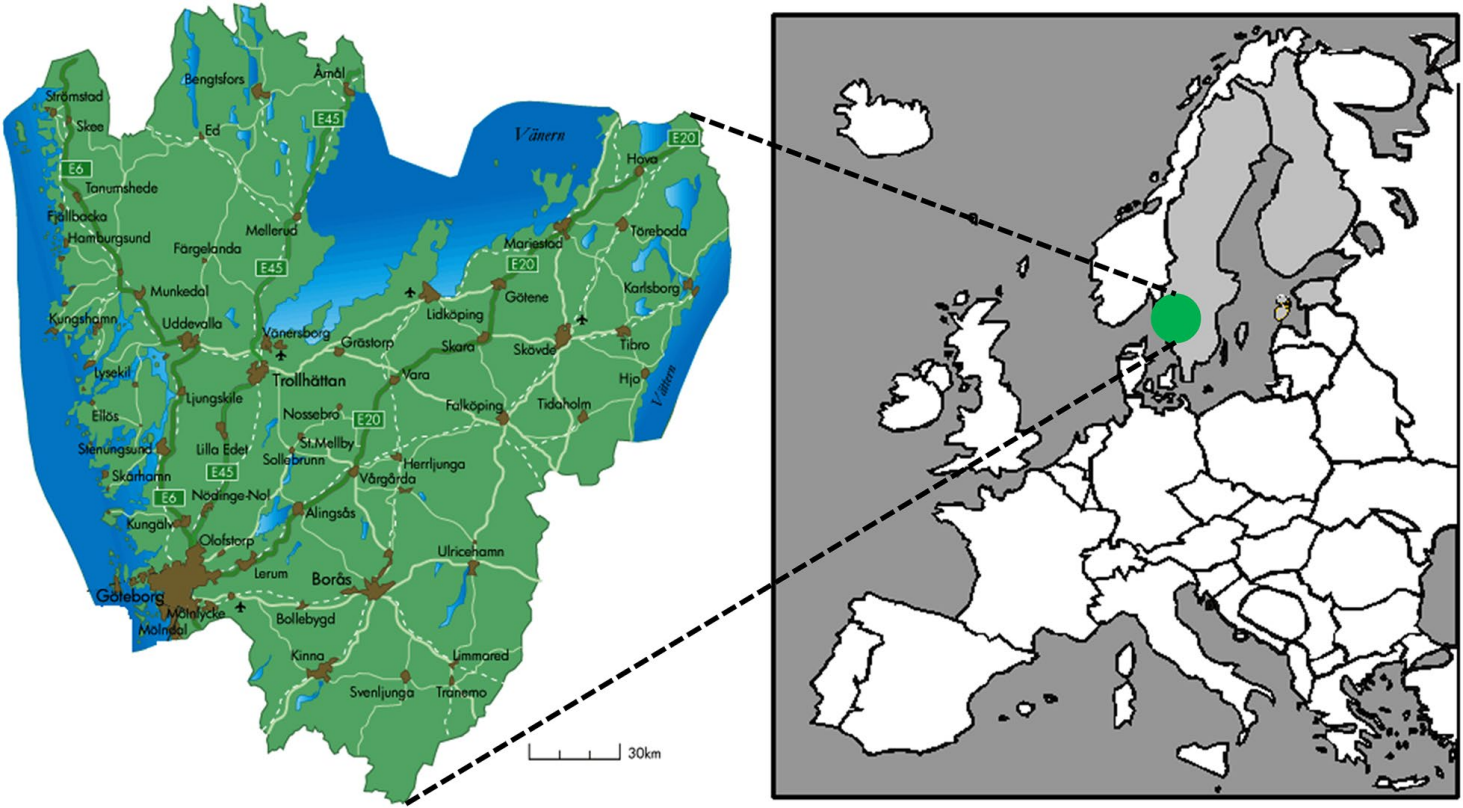
Map of Sweden depicting the geographic area of West Sweden where the study was conducted

**Fig. 2:**
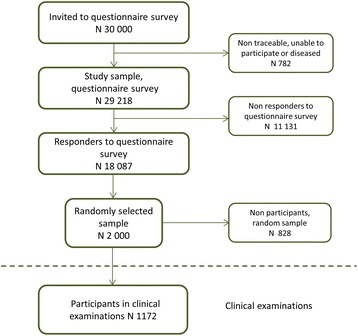
Flowchart of the study design and participation

The West Gothia region of Sweden (Fig. 1) has a population of 1.6 million, approximately one-sixth of the total Swedish population. In 2008, a large population based questionnaire survey was dispatched by mail to 30,000 randomly selected subjects in this area. From the metropolitan area of Gothenburg 15,000 subjects were selected and 15,000 subjects from the rest of the county. Response rate was 62 % (18,087 participants). The population study and a study on effects of late and non-response has previously been described in detail [18, 19]. Among the responders, 2000 randomly selected participants were invited to clinical examinations and 1172 (59 %) individuals participated. Figure 2 shows the study set-up and participation.

### Questionnaire and clinical examinations

The questionnaire consisted of three parts administered at the same time. The first part was the Swedish OLIN study questionnaire [20, 21] covering asthma, rhinitis, COPD, respiratory symptoms and possible risk factors of disease such as smoking and family history of airway diseases. The second part included questions regarding occupational exposures, environmental exposures and health status. The third part consisted of the Swedish Global Allergy and Asthma (GA^2^LEN) questionnaire [22] which further assessed respiratory symptoms and diseases and added questions about eczema. The clinical examinations included objective measures of height and weight, a structured interview and a drawn blood sample. Sixty-nine subjects did not participate in the blood sampling either due to unwillingness or technical difficulties but completed the other parts of the examination. The presence of serum Immunoglobulin E (IgE) was assessed using a mixture of 11 common airborne allergens and included timothy, birch, mugwort, olive, parietaria, cat, dog, horse, *D. pteronyssinus*, *D. farinae* and *C. herbarum* (ImmunoCAP^®^ Phadiatop, Thermo Scientific). A value of ≥0.35 kU_A_/l was considered a positive test. Body Mass Index (BMI) was calculated by weight_(kg)_/(height_(m)_)^2^. BMI was defined as normal if 20–25 kg/m^2^, underweight <20 kg/m^2^, overweight 25–30 kg/m^2^ and more than 30 kg/m^2^ considered as obesity.

### Definitions

*Asthma ever*: Either “Have you ever had asthma?” or “Have you ever been diagnosed as having asthma by a physician?”

*Current asthma*: *Asthma ever* and a report of either use of asthma medication or recurrent wheeze or attacks of shortness of breath in the last 12 months.

*Eczema ever*: “Have you ever had eczema or any kind of skin allergy?”

*Current eczema*: Yes to “Have you ever had an itchy rash which was coming and going for at least 6 months?” and “Have you had this itchy rash in the last 12 months?”

*Rhinitis ever*: Either “Have you ever had nasal allergies or hay fever?”, or *nasal blockage:* “Do you have nasal blockage more or less constantly?’, or *runny nose:* “Do you have a runny nose more or less constantly?”

*Current rhinitis*: Either *nasal blockage* or *runny nose,* or both of the following: “Have you had sneezing, runny nose or nasal blockage apart from colds during the last 12 months?” and “Have these nasal symptoms occurred simultaneously with itching and running eyes?”

*Smoking*: Smokers reported smoking the year preceding the study and ex-smokers reported having quit smoking at least 1 year before the study. Non-smokers reported neither smoking nor ex-smoking.

*Allergic sensitization*: Serum IgE to ImmunoCAP^®^ Phadiatop ≥0.35 kU_A_/l

*Occupational exposure to gas, dust or fumes* (GDF): “Have you been substantially exposed to dust, gases or fumes at work?”

*Raised on a farm*: “Did your family live on a farm during your first 5 years of life?”

*Childhood airway infection*: “Have you had any severe airway infection or pneumonia before school age, such as whooping cough or croup?”

*Childhood daycare*: “Did you attend preschool, day care or an orphanage with other children for at least 1 year before school age?”

### Analyses

Statistical analyses were performed using IBM SPSS version 20.0.0. The significance level was set to 0.05. Tests for in group differences in proportions were calculated with Fisher’s exact test. Tests for trend were computed with a Mantel–Haenszel test where appropriate. Pearson Chi^2^ was performed for in group differences in contingency tables larger than two by two. Crude Odds ratios (OR) and 95 % confidence intervals (CI) were calculated for the variables current asthma, current rhinitis and current eczema. Models of multiple logistic regressions were calculated with adjusted ORs and 95 % CIs. Independent covariates were: age divided in three categories, sex, family history of asthma and rhinitis, exposure to gas, dust and fumes at work, raised on a farm, BMI, allergic sensitization, number of siblings and childhood daycare.

## Results

### Prevalence of asthma, rhinitis, eczema and risk factors

Mean age ± SD of the participants was 50.4 ± 15.4 years with 54 % women. The prevalence of current asthma was 11.8 %, current rhinitis 42.8 % and current eczema 13.5 % (Table 1). Prevalence of asthma ever, eczema ever, rhinitis ever and current rhinitis were all inversely associated with increasing age, as was family history of asthma and rhinitis. Eczema was more common among women and women were also more likely to report a family history of asthma or rhinitis. Usage of rhinitis medications in the last 12 months was more common among younger subjects (P < 0.001). BMI increased by age. Men tended to have a higher BMI and were more exposed to gas, dust or fumes at work compared to women. The prevalence of allergic sensitization was 29.7 %, decreased by age and was more common among men (Table 1).

**Table 1:** Baseline characteristics of the studied population

	Age (years)				Sex			
	18–39	40–59	60–77	P value^1^	Men	Women	P value^2^	Total (95 % CI)
Current asthma	12.3	13.4	9.4	0.216	10.1	13.2	0.122	11.8 (9.9–13.6)
Asthma ever	17.8	16.2	11.8	0.024	14.0	16.2	0.328	15.2 (13.1–17.3)
Current rhinitis	48.8	43.8	36.6	0.001	44.5	41.4	0.314	42.8 (40.0–45.7)
Rhinitis ever	53.7	48.5	39.5	<0.001	50.0	44.4	0.060	47.0 (44.2–50.0)
Current eczema	12.9	17.0	9.7	0.167	11.3	15.4	0.040	13.5 (11.5–15.4)
Eczema ever	49.1	46.8	38.2	0.003	36.5	51.6	<0.001	44.6 (41.8–47.5)
Asthma medication 12 m	11.0	12.5	10.7	0.864	9.8	13.0	0.098	11.5 (9.7–13.4)
Rhinitis medication 12 m	33.7	30.8	19.9	<0.001	27.1	28.9	0.515	28.1 (25.5–30.6)
Allergic sensitization	40.9	31.0	18.6	<0.001	36.0	24.2	<0.001	29.7 (27.0–32.4)
*Smoking*								
Never smoker	70.6	52.1	49.5	<0.001^*3*^	58.5	54.5	0.015^*3*^	56.4 (53.5–59.2)
Ex-smoker	11.0	29.7	37.6		28.3	26.0		27.1 (24.5–29.6)
Current smoker	18.4	18.2	12.9		13.1	19.5		16.5 (14.4–18.7)
*Family history*								
Asthma	23.8	20.2	15.0	0.004	15.8	22.6	0.004	19.5 (17.2–21.8)
Rhinitis	42.9	35.2	14.0	<0.001	25.3	35.3	<0.001	30.6 (27.9–33.4)
Asthma and rhinitis	16.7	12.6	4.7	<0.001	7.9	14.1	0.002	11.2 (9.4–13.1)
Asthma or rhinitis	49.8	42.1	24.0	<0.001	32.9	43.1	<0.001	38.4 (35.6–41.2)
*Body mass index*								
Underweight (<20)	10.4	3.0	1.0	<0.001^*3*^	1.5	7.0	<0.001^*3*^	4.4 (3.3–5.6)
Normal (20–25)	54.9	34.7	28.0		33.2	42.4		38.1 (35.4–40.9)
Overweight (25–30)	26.1	45.0	52.4		50.7	34.8		42.2 (39.3–45.0)
Obese (>30)	8.6	17.2	18.6		14.6	15.9		15.3 (13.2–17.3)
Occupational GDF	24.3	24.6	25.3	0.750	36.2	14.9	<0.001	24.7 (22.3–27.2)
Metropolitan domicile	70.6	50.9	52.4	<0.001	54.8	58.6	0.214	57.0 (54.0–60.0)
Raised on a farm	7.7	11.8	19.9	<0.001	13.9	12.8	0.604	13.3 (11.4–15.3)

Prevalence (%) and difference (*p* value) by sex and age group. Total prevalence with 95 % confidence intervals (95 % CI)

^1^Test for trend

^2^Fishers exact two sided test

^3^Pearson chi^2^ test for in group difference

**Fig. 3:**
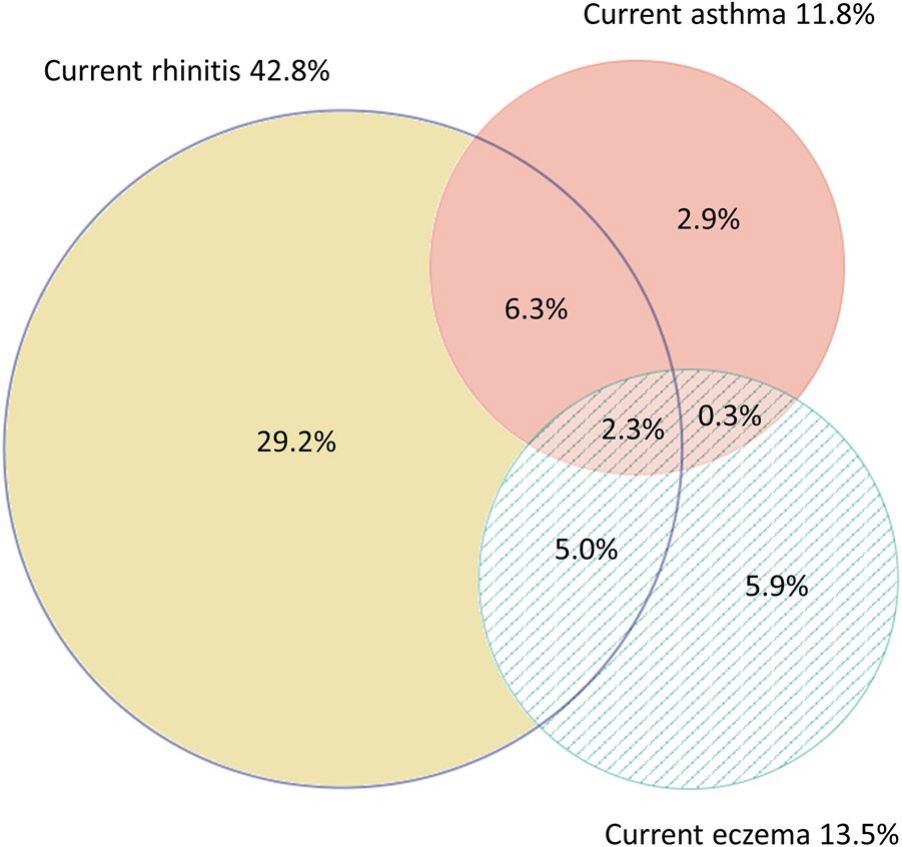
Circular Venn diagram illustrating the prevalence of current rhinitis, current asthma and current eczema; and the overlap between the conditions

Figure 3 show the relationship between current asthma, current rhinitis and current eczema. Of all subjects, a similar proportion had both current asthma and current rhinitis as current eczema and current rhinitis, 8.5 % and 7.3 % respectively, while 2.6 % had current asthma and current eczema. Only 2.3 % had all three conditions and current rhinitis was the most common single condition (Fig. 3). The relationship between asthma ever, rhinitis ever and eczema ever showed a similar pattern and is available in the appendix (Additional file 1: Figure S1).

### Unadjusted risk factors for asthma, rhinitis and eczema

Table 2 shows unadjusted associations for the conditions expressed as odds ratios. Younger age was a significant risk factor for current rhinitis but not for current asthma. For current eczema, only the 40–59 years of age category had an increased risk compared to the oldest. Female sex only increased the risk of current eczema. Family history of both asthma and rhinitis had the strongest positive association with current asthma (OR 5.4 CI 3.4–8.6) but also significantly increased the risk for current rhinitis and current eczema. Allergic sensitization was a strong risk factor for both current asthma (OR 4.0 CI 2.8–5.4) and current rhinitis (OR 5.8 CI 4.3–7.7) but not significantly so for current eczema (OR 1.4 CI 0.9–1.9). Exposure to gas, dust or fumes at work was a significant risk factor for both current eczema and current asthma (both OR 1.7). Obesity was associated with an increased risk of current rhinitis and current asthma but not of current eczema, while degree of urbanization and metropolitan domicile was not associated with any of the three conditions.

**Table 2:** Crude odds ratios (OR) and 95 % confidence intervals (CI) for current asthma, current rhinitis and current eczema

Independent variables	Dependent variables		
	Current asthma	Current rhinitis	Current eczema
	OR (95 % CI)	OR (95 % CI)	OR (95 % CI)
*Age (years)*			
60–77	1	1	1
40–59	1.48 (0.96–2.29)	1.34 (1.02–1.77)	1.91 (1.26–2.90)
18–39	1.34 (0.83–2.17)	1.65 (1.22–2.22)	1.38 (0.86–2.20)
*Sex*			
Men	1	1	1
Women	1.34 (0.94–1.93)	0.88 (0.70–1.11)	1.45 (1.05–2.00)
*Smoking*			
Never smoker	1	1	1
Ex-smoker	0.86 (0.56–1.31)	0.96 (0.73–1.26)	1.28 (0.87–1.89)
Current smoker	1.04 (0.64–1.70)	1.15 (0.83–1.59)	1.46 (0.93–2.28)
*Family history*			
None	1	1	1
Asthma	1.33 (0.58–3.05)	0.72 (0.42–1.25)	1.44 (0.72–2.85)
Rhinitis	1.49 (0.88–2.51)	3.05 (2.20–4.24)	1.32 (0.83–2.08)
Both	5.41 (3.38–8.64)	3.32 (2.23–4.95)	1.82 (1.10–3.00)
*Degree of urbanization*			
Rural area (<500 inh.)	1	1	1
Village (500–2000 inh.)	0.88 (0.29–2.65)	1.23 (0.65–2.37)	0.97 (0.35–2.74)
Small town (2000–10,000 inh.)	1.28 (0.55–3.00)	1.01 (0.58–1.75)	1.16 (0.51–2.68)
Larger town (>10,000 inh.)	1.29 (0.67–2.48)	1.41 (0.94–2.11)	1.39 (0.74–2.61)
Occupational exposure to GDF			
No	1	1	1
Yes	1.70 (1.16–2.49)	1.10 (0.84–1.44)	1.73 (1.21–2.48)
*Metropolitan domicile*			
No	1	1	1
Yes	0.86 (0.60–1.23)	1.03 (0.81–1.30)	1.21 (0.86–1.70)
*Body mass index*			
Normal	1	1	1
Underweight	1.17 (0.47–2.88)	1.34 (0.75–2.38)	1.24 (0.56–2.78)
Overweight	1.07 (0.71–1.63)	0.94 (0.73–1.22)	0.98 (0.67–1.44)
Obese	2.10 (1.29–3.40)	1.79 (1.26–2.53)	1.43 (0.89–2.31)
*Allergic sensitization*			
No	1	1	1
Yes	4.02 (2.76–5.84)	5.77 (4.34–7.67)	1.35 (0.94–1.93)

Subjects that grew up on a farm had a lower risk of both current eczema and current rhinitis (OR 0.5 CI 0.2–0.9 and 0.5 CI 0.4–0.8) respectively (Table 3). Childhood smoke exposure and maternal smoking in pregnancy was associated with an increased risk of current eczema, while current smoking status was not significantly associated with any of the three conditions. Childhood airway infection increased the risk of asthma. Having one or more sibling increased the risk of current asthma whereas childhood daycare increased the risk of current eczema. Birth weight and educational level were not associated with any of the three conditions (Table 3). Risk factors for eczema ever, asthma ever and rhinitis ever were similar with a few exceptions; most notably allergic sensitization was also positively associated with eczema ever (Additional file 2: Table S1).

**Table 3:** Crude odds ratios (OR) and 95 % confidence intervals (CI) for current asthma, current rhinitis and current eczema

Independent variables	Dependent variables		
	Current asthma	Current rhinitis	Current eczema
	OR (95 % CI)	OR (95 % CI)	OR (95 % CI)
*Raised on a farm*			
No	1	1	1
Yes	0.84 (0.48–1.45)	0.54 (0.38–0.78)	0.45 (0.24–0.85)
*Number of siblings*			
0	1	1	1
1	3.06 (1.29–7.25)	1.37 (0.92–2.06)	0.76 (0.45–1.29)
2 or more	2.76 (1.17–6.49)	1.32 (0.89–1.97)	0.59 (0.35–0.99)
*Childhood daycare*			
No	1	1	1
Yes	1.14 (0.80–1.63)	1.40 (1.11–1.77)	1.48 (1.06–2.07)
*Childhood smoke exposure*			
No	1	1	1
Yes	1.05 (0.73–1.50)	0.91 (0.72–1.15)	1.52 (1.06–2.16)
*Maternal smoking in pregnancy*			
No	1	1	1
Yes	0.70 (0.35–1.37)	1.04 (0.70–1.54)	1.89 (1.13–3.14)
*Furry animals in childhood*			
Yes	1	1	1
No	1.19 (0.83–1.70)	1.05 (0.75–1.49)	1.24 (0.98–1.57)
*Childhood airway infection*			
No	1	1	1
Yes	2.35 (1.57–3.51)	0.94 (0.73–1.23)	1.34 (0.92–1.94)
*Shared bedroom in childhood*			
No	1	1	1
Yes	1.22 (0.85–1.76)	0.91 (0.72–1.16)	0.99 (0.70–1.39)
*Birth weight*			
3000–4000 g	1	1	1
Less than 2500 g	2.00 (0.89–4.50)	1.22 (0.65–2.31)	0.96 (0.39–2.35)
2500–3000 g	1.41 (0.81–2.45)	1.04 (0.71–1.52)	1.21 (0.73–1.99)
More than 4000 g	0.72 (0.36–1.44)	0.89 (0.60–1.31)	0.54 (0.28–1.04)
*Level of education*			
Secondary school or less	1	1	1
High school	1.00 (0.59–1.67)	1.10 (0.79–1.54)	1.49 (0.91–2.43)
University	1.09 (0.67–1.78)	1.15 (0.84–1.58)	1.13 (0.69–1.84)

### Adjusted risk factors for asthma, rhinitis and eczema

Age was not a risk factor for any of the conditions when adjusted odds ratios were calculated in multiple regression models (Table 4). Female sex was a risk factor for both current asthma (OR 1.8 CI 1.2–2.7) and current eczema (OR 1.7 CI 1.2–2.5). Family history of asthma and rhinitis significantly increased the risk of current rhinitis, and the risk of current asthma was of an even greater magnitude, while it was not significantly associated with current eczema. Allergic sensitization was the strongest risk factor for current rhinitis (OR 5.1 CI 3.8–6.9) and the second strongest for current asthma (OR 4.1 2.7–6.3) but was not associated with current eczema.

**Table 4:** Adjusted risk factors for current asthma, current rhinitis and current eczema

Independent variables	Dependent variables		
	Current asthma	Current rhinitis	Current eczema
	OR (95 % CI)	OR (95 % CI)	OR (95 % CI)
*Age (years)*			
60–77	1	1	1
40–59	1.00 (0.61–1.63)	0.88 (0.64–1.23)	1.51 (0.95–2.38)
18–39	0.73 (0.39–1.37)	0.92 (0.61–1.41)	0.88 (0.49–1.57)
*Sex*			
Men	1	1	1
Women	1.77 (1.15–2.70)	0.91 (0.69–1.21)	1.71 (1.17–2.51)
*Family history*			
None	1	1	1
Asthma	1.29 (0.55–3.04)	0.61 (0.34–1.10)	1.45 (0.72–2.94)
Rhinitis	1.16 (0.66–2.03)	2.58 (1.79–3.72)	1.25 (0.77–2.04)
Both	4.33 (2.57–7.30)	2.76 (1.77–4.31)	1.61 (0.94–2.76)
*Occupational exposure to GDF*			
No	1	1	1
Yes	1.85 (1.20–2.87)	1.03 (0.76–1.41)	2.08 (1.40–3.08)
*Raised on a farm*			
No	1	1	1
Yes	0.96 (0.52–1.77)	0.64 (0.42–0.96)	0.51 (0.27–0.99)
*Body mass index*			
Normal	1	1	1
Underweight	0.95 (0.36–2.55)	1.42 (0.74–2.71)	1.00 (0.44–2.30)
Overweight	1.04 (0.65–1.67)	1.04 (0.77–1.41)	1.02 (0.68–1.54)
Obese	1.95 (1.13–3.36)	2.30 (1.53–3.46)	1.34 (0.80–2.24)
*Allergic sensitization*			
No	1	1	1
Yes	4.11 (2.71–6.25)	5.11 (3.77–6.93)	1.26 (0.85–1.85)
*Number of siblings*			
0	1	1	1
1	3.09 (1.25–7.63)	1.16 (0.74–1.81)	0.72 (0.42–1.24)
2 or more	2.51 (1.02–6.15)	1.15 (0.74–1.79)	0.55 (0.32–0.94)
*Childhood daycare*			
No	1	1	1
Yes	0.92 (0.59–1.44)	1.11 (0.81–1.52)	1.40 (0.94–2.10)

Risk is expressed in odds ratios (OR) with 95 % confidence intervals (CI)

Exposure to gas dust or fumes as work remained a stable risk factor for current eczema and current asthma after adjustment. Growing up on a farm remained similarly associated with a protective effect on current eczema (OR 0.5 CI 0.3–0.99) and current rhinitis (OR 0.6 CI 0.4–0.96). Obesity was a risk factor for current asthma and current rhinitis but not for current eczema. Having siblings was a risk factor for current asthma but childhood daycare showed no effect in the multivariate models. Maternal smoking in pregnancy, childhood smoke exposure and smoking status were added separately to the model but were not significantly associated with any of the conditions and did not alter the other risk estimates (not shown). Childhood airway infection was a risk factor for current asthma, OR 2.6 (95 % CI 1.7–4.1) when added separately to the models but did not alter the magnitude of the other risk factors. An additional multivariate analysis was performed to assess the risk factor pattern of subjects with the combination of current asthma and current rhinitis. The results showed that the risk factor pattern in this group was close to identical to subjects with current asthma (data not shown).

Adjusted odds ratios for eczema ever, rhinitis ever and asthma ever showed similar results for most risk factors with some exceptions. Most notably, exposure to gas dust or fumes was not a risk factor for any of the conditions and growing up on a farm had no significant protective on rhinitis ever and eczema ever. However, allergic sensitization was a risk factor for all of eczema ever, rhinitis ever and asthma ever (Additional file 3: Table S2).

## Discussion

### Main findings

In this population based study of 1172 adults we found that family history of both asthma and allergy was the strongest risk factor for current asthma and it also increased the risk of current rhinitis but not for current eczema after adjustment. Allergic sensitization was an important risk factor for current asthma and current rhinitis, but for not current eczema, and obesity showed the same pattern. Occupational exposure to gas, dust and fumes was positively associated with both current asthma and current eczema but not with current rhinitis. Growing up on a farm had a protective effect on both current eczema and current rhinitis but not on current asthma. Female sex was associated with an increased risk for current asthma and current eczema but not for current rhinitis.

### Comparisons to other studies

Comparisons with other studies should be interpreted with caution due to varying definitions of disease. The prevalence of current eczema in our study was 13.5 %. Earlier studies addressing eczema in adults have found similar magnitudes of prevalence with 11.6 % in Sweden [23], 8.1 % in Italy [24], 14.3 % in Denmark [25], 11.5 % in Colombia [26] and 10.2–10.7 % in the United States [16, 17]. Earlier studies of rhinitis have mainly focused on either allergic rhinitis or symptoms of chronic rhinosinusitis. Our definition of current rhinitis included subjects with either allergic rhinitis or other chronic nasal symptoms. The observed prevalence of current rhinitis in our study was 42.8 %. This high prevalence of rhinitis may seem remarkable; however, another recent study in Sweden found that the prevalence of rhinitis symptoms was 51 % [27]. Further, 28.1 % in our study reported use of medication against rhinitis in the last year. Allergic rhinitis has also seen a significant increase in prevalence during the last thirty years [28–30]. The prevalence in Italy has been estimated at 26 % [31] and in southern Finland it has exceeded 40 % [32].

The prevalence of current asthma was 11.8 %. This is of a similar magnitude to Australia where current asthma was present in 8.3–13.1 % depending on age and sex [33], and to Finland where 10.0 % reported asthma and an absolute majority also had symptoms [34]. Other studies have found lower prevalence with 6.6 % in Italy [31] and from 7 to 11 % in Denmark [35, 36]. Noteworthy, all of those studies did not involve older adults and had a more narrow definition of asthma with the Italian study excluding subjects not using medication against asthma.

In agreement with earlier studies [37] we found allergic sensitization to be a strong risk factor for current asthma and current rhinitis. Studies have established that allergic sensitization is a risk factor for childhood eczema [38] but published results among adults are scarce. One pooled study from Europe and the United States [39] found a weak positive association between allergic sensitization and eczema while a stronger association was found in a Danish study [40]. That our study did not show a significant association between eczema and allergic sensitization may reflect a weaker association in adults compared to children, yet also be attributed to the definition of eczema where studies presenting a stronger association often employ traditional definitions of eczema that are affected by asthma and rhinitis [41].

The finding that current asthma and current rhinitis are associated to family histories of those conditions is well known [42, 43], yet the impact of family history of other atopic diseases on adult eczema has not been fully established. One of very few studies on this subject did show an association [44], while in our study no association to either family history of asthma, family history of rhinitis or both was noted. The finding that more women than men suffer from current asthma is in agreement with earlier results [45, 46]. We could also replicate the positive association between female sex and current eczema that previously has been demonstrated in the few studies on this topic [15, 44]. The cause for this gender related difference is largely unknown but different expression of sex hormones may play a role at least for asthma [47].

Occupational exposure to gas, dust or fumes increased the risk for both current asthma and current eczema. In the case of asthma, this has previously been reported [48, 49] and it has been estimated that 14 % of adult onset asthma may be attributed to occupational exposure to GDF [50]. The effect of occupational exposure to GDF on eczema is relatively unknown because most studies on eczema to this date have focused on children. Earlier occupational studies regarding eczema have been limited to contact dermatitis. There are reports highlighting the importance of airborne particles for eczema. A Turkish study [51] found that using wood for house heating was associated with eczema, and in Germany [52] there was an increase in eczema after a large accidental airborne emission of chemicals.

Occupational agents can cause asthma by acting as antigens resulting in an IgE mediated allergic reaction and can also exert a direct effect on the respiratory epithelium with subsequent cell-mediated inflammation that is independent of IgE [53]. The mechanism by how airborne particles from an occupational setting may cause eczema is yet to be elucidated. Recent work with the skin the barrier disruption in focus has gained attention [54] which may explain a plausible mechanism for the role between eczema and occupational exposure.

We found that having lived on a farm during the first 5 years of life was associated with a protective effect on both current rhinitis and current eczema but not current asthma. This contrasts results from studies among children where a protective association with asthma has been shown [55]. The protective effect of farm childhood on rhinitis has been observed in young adults in Finland [56] Germany [57] and Sweden [43]. However, the association between farm living and eczema has not been found in populations of children [58]. In older subjects, a protective effect has been shown in Swedish military conscripts [59]. This is, to our knowledge, the first population based epidemiologic study showing a significant protective association between farm childhood and current eczema in adults.

Our observed positive association between obesity and current asthma has already been established in both cross-sectional [60, 61], case-referent [62] and longitudinal studies [63, 64]. We also found that current rhinitis, but not current eczema was positively associated with obesity. The effect of obesity on rhinitis and eczema is so far infrequently studied with conflicting results. In Japan, a study found that allergic rhinitis was associated with obesity only in conjunction with asthma or wheeze [65]. Another study in Poland found that asthma but not eczema or rhinitis was associated with obesity [66]. However, obese subjects in the United States were more likely to report eczema [15, 67].

### Strengths and limitations

There are several methodological strengths and weaknesses with cross-sectional studies of this type. The strength of the randomized population based approach of our study is that it decreases the risk of selection bias. Further, a non-response study comparing the responders to the non-responders of the postal survey has been carried out that did not show any significant differences in terms of diseases or symptoms [19]. While a participation rate of 59 % in the clinical examinations is satisfying and comparable to other international studies, there is a risk of bias with enrichment of subjects with the condition studied. An analysis was performed that compared participants and the non-participants in the clinical examination. No differences were seen in gender, smoking or reported use of asthma medication. However, prevalence was slightly higher for asthma and rhinitis but we do not believe that this had any major influence on the studied associations. The questions used in the survey were from internationally validated questionnaires and anthropometric parameters were objectively measured. The cross-sectional design of the study has a weakness in its inability to infer causality between the dependent and the independent variables. Associations must thus be regarded with uncertain causality. Recall bias is a concern where for an example individuals with asthma may be better informed of childhood infections compared to healthy subjects. Another weakness is that the questions on eczema may include other non-eczematous types of dermatitis. Validation studies on questions about eczema similar to ours in children have shown good sensitivity but lower specificity [68].

## Conclusions

We conclude that there are different risk factor patterns for asthma, rhinitis and eczema among adults. No common risk factor was identified for all three entities. However, some exposures and covariates, such as obesity, farm childhood and allergic sensitization are risk factors for two of the conditions but not all three. Future epidemiological research on the combined determinants of the diseases is needed and especially for rhinitis and eczema.

## Additional files

10.1186/s13601-016-0112-0  Circular Venn diagram illustrating the prevalence of rhinitis ever, asthma ever and eczema ever; and the overlap between the conditions. (Filename “Additional file 1: Figure S1.png”, File format Portable Network Graphics).
10.1186/s13601-016-0112-0 Crude odds ratios (OR) and 95 % confidence intervals (CI) for asthma ever, rhinitis ever and eczema ever. (Filename “Additional file 2: Table S1.xlsx”, File format Microsoft Excel Worksheet).
10.1186/s13601-016-0112-0 Adjusted risk factors for asthma ever, rhinitis ever and eczema ever. Risk is expressed in odds ratios (OR) with 95 % confidence intervals (CI). (Filename “Additional file 3: Table S2.xlsx”, File format Microsoft Excel Worksheet).

## Abbreviations

BMI: body mass index
CI: confidence interval
GA^2^LEN: Swedish Global Allergy and Asthma
GDF: occupational exposure to gas, dust or fumes
OR: odds ratio
