# Supplementary figures and images for: Different risk factor patterns for adult asthma, rhinitis and eczema: results from West Sweden Asthma Study
Source: Clin Transl Allergy. 2016 Aug 4;6:28. doi: 10.1186/s13601-016-0112-0 (PMC4973051; doi:10.1186/s13601-016-0112-0)

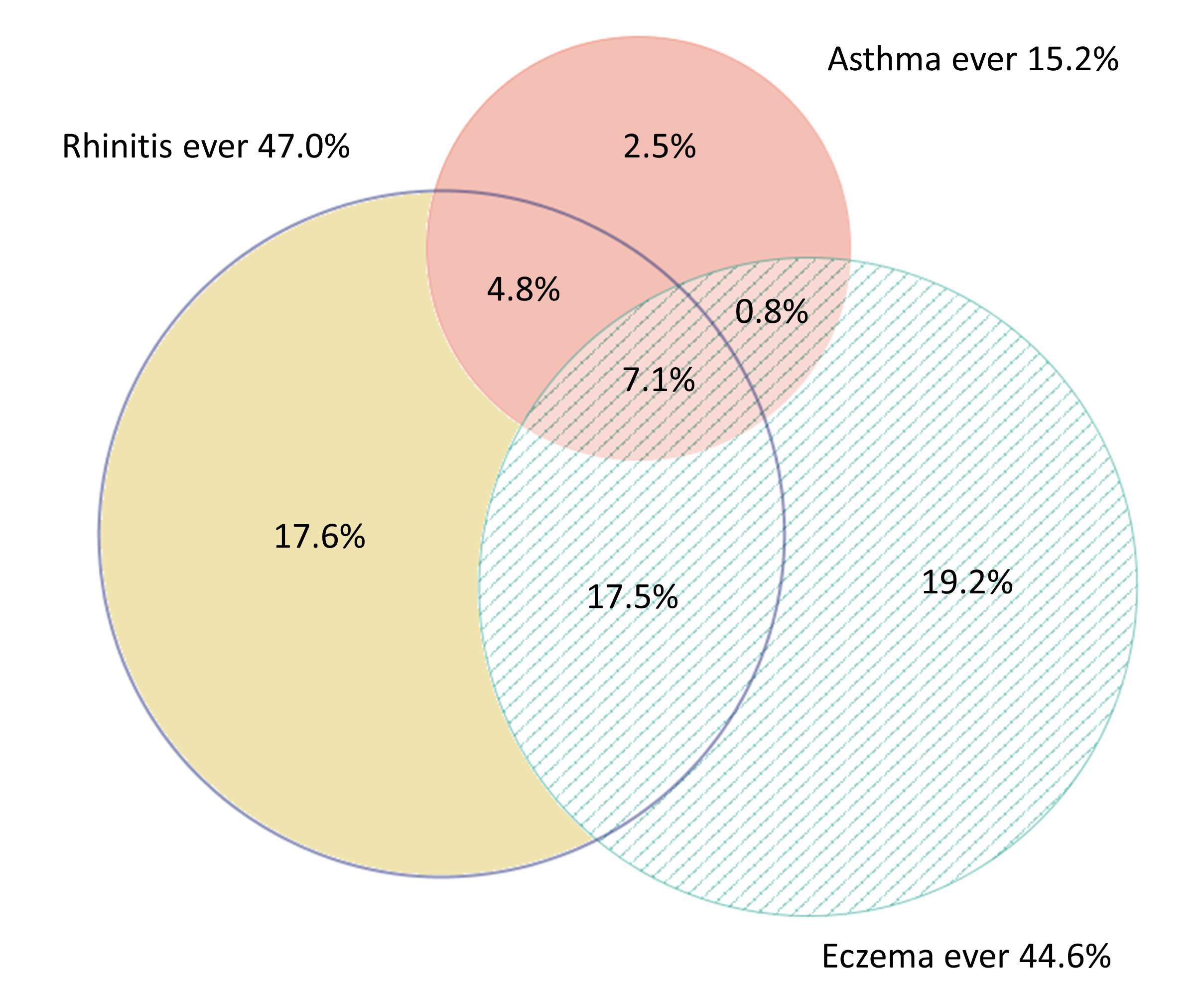

Supplement: Supplementary file 1 — 10.1186/s13601-016-0112-0 Circular Venn diagram illustrating the prevalence of rhinitis ever, asthma ever and eczema ever; and the overlap between the conditions. (Filename “Additional file 1: Figure S1.png”, File format Portable Network Graphics). [file 13601_2016_112_MOESM1_ESM.png]
